# A quantitative characterization of interaction between prion protein with nucleic acids

**DOI:** 10.1016/j.bbrep.2018.04.006

**Published:** 2018-05-02

**Authors:** Alakesh Bera, Sajal Biring

**Affiliations:** a Infectiologie Animale et Santé Publique, Institut National de la Recherche Agronomique, 37380 Nouzilly, France; b Department of Electronic Engineering and Organic Electronics Research Center, Ming-Chi University of Technology, 84 Gungjuan Rd., Taishan Dist., New Taipei City 24301, Taiwan

**Keywords:** Prion protein, Small highly structured RNAs (shsRNAs), Pseudoknots, Poly A, Fluorescence anisotropy (r), Binding constant (Kd)

## Abstract

Binding of recombinant prion protein with small highly structured RNAs, prokaryotic and eukaryotic prion protein mRNA pseudoknots, tRNA and polyA has been studied by the change in fluorescence anisotropy of the intrinsic tryptophan groups of the protein. The affinities of these RNAs to the prion protein and the number of sites where the protein binds to the nucleic acids do not vary appreciably although the RNAs have very different compositions and structures. The binding parameters do not depend upon pH of the solution and show a poor co-operativity. The reactants form larger nucleoprotein complexes at pH 5 compared to that at neutral pH. The electrostatic force between the protein and nucleic acids dominates the binding interaction at neutral pH. In contrast, nucleic acid interaction with the incipient nonpolar groups exposed from the structured region of the prion protein dominates the reaction at pH 5. Prion protein of a particular species forms larger complexes with prion protein mRNA pseudoknots of the same species. The structure of the pseudoknots and not their base sequences probably dominates their interaction with prion protein. Possibilities of the conversion of the prion protein to its infectious form in the cytoplasm by nucleic acids have been discussed.

## Highlights

• Species specific bindings occur between prion protein and its mRNA pseudoknots.
• Interactions between shsRNAs and prion protein are modulated with their structure.
• Formation of RNA-prion protein complexes are pH dependent.
• Biophysical analysis delineates the RNA-prion protein binding parameters.

## 1 Introduction

Cellular prion protein, PrP^**C**^, is a soluble α-helix rich glycoprotein attached to the outer cell surface by a glycophosphatidyl inositol linkage [1], [2]. The biological role of PrP^**C**^ remains mostly unknown but the protein has been suggested to play different roles including maintain the cellular copper concentration, different signal transduction, RNA binding, and DNA metabolism [3], [4], [5], [6]. The protein is non-infectious but its β-sheet rich isoform, PrP^Sc^, is considered as the major infectious component for the genetic, sporadic as well as transmissible fatal neurodegenerative prion diseases [1], [2]. It has been demonstrated that structural conversion of the cellular prion protein to its scrapie isoform PrP^**Sc**^ takes place in acidic pH5 in the endosomes and lysosomes [7], [8], [9], [10], [11]. PrP^**Sc**^ can exist as oligomers or insoluble amyloid polymers and is resistant to Proteinase K (PK) digestion whereas PrP^**C**^ is digested by the PK enzyme [1], [2].

Unlike bacterial and viral diseases where nucleic acid transmits the infection, prion disease has been considered to propagate by the conversion of PrP^**C**^ to PrP^**Sc**^, which can occur either by a template or a nucleation mechanism [1], [2]. The existence of multiple prion strains has also been attributed to the conformational variations of PrP^**Sc**^ although the existence of a nucleic acid as a cofactor for infection can explain the strain multiplicity [12], [13], [14], [15]. The propagation of non-neuronal PrP^**Sc**^ in the experimental mice has been found to be non-pathogenic and arresting the conversion of PrP^**C**^ to PrP^**Sc**^ within neurons during prion infection has been found to prevent prion neurotoxicity [16]. The fibrils formed from in vivo isolated hamster PrP 27–30 amyloid or fibrils obtained by converting cellular hamster PrP^**C**^ have been found to be noninfectious in transgenic mice over-expressing full-length Syrian prion protein [17]. However, the amyloid formed from the truncated 90–231 fragment of mouse recombinant prion protein (23–231 amino acid) is found to be infectious in the experimental mice over-expressing this protein fragment and also shows strain characteristics of the prion disease [18], [19]. Inoculation of wild-type hamsters with in vitro-generated PK-resistant prion protein formed by protein misfolding cyclic amplification has been found to be infectious [20]. By partially disaggregating PK-resistant amyloid isolated from scrapie infected hamster brain, it has been shown that the maximum prion infectivity is associated with prion particles having 17–27 nm diameter (300–600 kDa) whereas the large fibrils show lower prion infectivity [21].

A number of different molecules have been found to facilitate conversion of prion protein to insoluble aggregates [22], [23], [24]. Our previous studies also indicated that the osmolyte trimethylamine N-oxide converts recombinant prion protein to its soluble beta-structured form at high temperature [25]. Besides, the synthetic nucleic acids, both in solution and in vitro, can catalyze conversion of recombinant and cellular PrP^**C**^ to PrP^**Sc**^ as evidenced from secondary structural studies of the protein and PK resistance properties [26], [27], [28], [29], [30]. The highly structured small RNA (shsRNAs) binds to PrP^**C**^ at neutral pH which yields Proteinase K resistant component in the presence of other cellular cofactors [28]. Another study based on using brain tissues have shown that an endogenous 300 nucleotide long RNA (100 kDa) can convert PrP^**C**^ to Protienase K resistant form in vitro [29]. These results indicate that nucleic acid can act as a cofactor for the conversion of PrP^**C**^ to PrP^**Sc**^ and can be the TSE mediator. In addition, multiple studies also indicated that the interaction between recombinant PrP^**C**^ and nucleic acids simultaneously produces a mixture of condensed and functionally active nucleoprotein complex, as well as PrP^Sc^ like oligomers and linear and spherical amyloids [6], [27], [31], [32]. To date, a specific nucleic acid as a cofactor for the propagation of prion infection has not been identified [33]. A recent study indicated that the lipid and RNA act as a cofactor for the recombinant prion protein to form PrP^Sc^ -like signature but lacks in vivo infectivity [34]. PrP^C^ is a cell surface protein, and nucleic acids in extra-cellular circulation can interact with it [6]. However, it has been considered that the relevant nucleic acid mediated PrP^**C**^ conversion towards its pathogenic form would be of cytoplasmic origin [6], [26], [27], [29], [31]. The presence of prion protein in cytoplasm of cells including neurons has been shown, and the exact biological role of prion protein-nucleic acid interaction is not known at present. However, it is hypothesized that the structural conversion of PrP^**C**^ to PrP^**Sc**^ can be catalyzed by cytoplasmic nucleic acids that can play a role in the prion diseases [35], [36], [37], [38]. Anti-prion activity of RNA aptamer reported as the RNA aptamers having preferential affinity to the PrP^Sc^ form [39]. These RNA aptamers also inhibit the conversion of PrP^C^ to the infectious form [40]. Besides, the small RNA drug is also suggested for prion disease [41].

At present no detailed study is available on the quantitative aspects of binding of the prion protein and nucleic acids, particularly RNAs. As mentioned above, small highly structured RNAs (shsRNA) bind to human recombinant prion protein with high affinity and specificity under physiological conditions demonstrated from gel electrophoresis studies [28]. These RNAs also can form highly stable nucleoprotein complexes with recombinant and cellular human prion protein (α-PrP) from various cell extracts and mammalian brain homogenates [28], [42].

The human prion protein gene contains five copies of a 24 nt repeat that is highly conserved among species [43], [44], [45]. Thermodynamic analyses of the repeat region suggest the presence of several hairpin loop structures and the presence of an RNA pseudoknot in human prion mRNA [43]. Computer generated three-dimensional structures of the human prion pseudoknot indicate prion protein and RNA interaction domains and the possible involvement in prion protein (PrP^C^) translation [43], [44], [45]. In the present investigation, we have studied the binding of shsRNAs and prion protein mRNA pseudoknots with human recombinant full-length prion protein and compared with binding properties of tRNA and poly A to the protein. A couple of studies have also been carried out with full-length mouse recombinant prion protein.

## 2 Materials and methods

### 2.1 Prion protein

The full length human and mouse recombinant prion proteins were isolated following the standard procedures [46], [47]. The human prion protein was a kind gift from Dr. H Rezaie (INRA, Jouy-en-Josas, France) ^45^. The mouse prion protein expression plasmid was a kind gift from Dr. R. Glockshuber [48]. The purity of both the proteins was over 95% as evidenced by polyacrylamide gel electrophoresis (PAGE) and mass spectrometry. Human prion protein concentration was calculated from the measured optical density at 280 nm applying an extinction coefficient value of 56795 M^−1^ Cm^−1^. Similarly, mouse prion protein concentration was calculated considering specific absorbance of 2.70 for 1 mg/ml at 280 nm.

### 2.2 Different RNA molecules

**Fig. 1:**
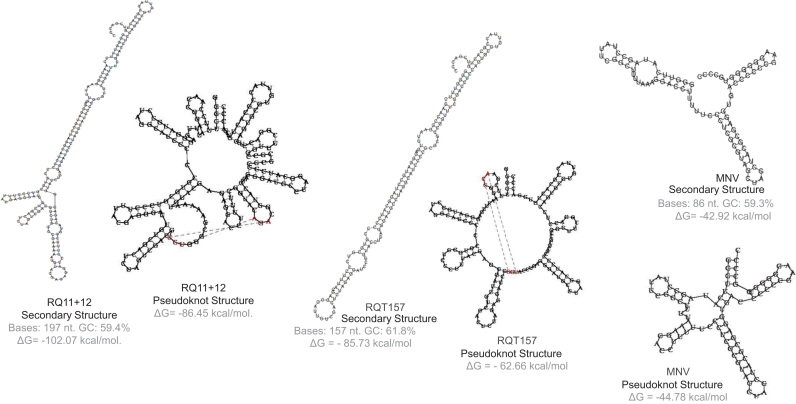
Structures of different small highly structured RNAs (shsRNAs) used in this study. The shsRNAs RQ11+12, RQT 157 and MNV contain 197, 157 and 86 nucleotides respectively. The sequence and other details are described in Table 1. The free-energy of formation and the secondary structures are made through *RNAstructure* website. The RNA pseudoknot structures are also projected through Heuristic Modeling *vsfold5*.

**Fig. 2:**
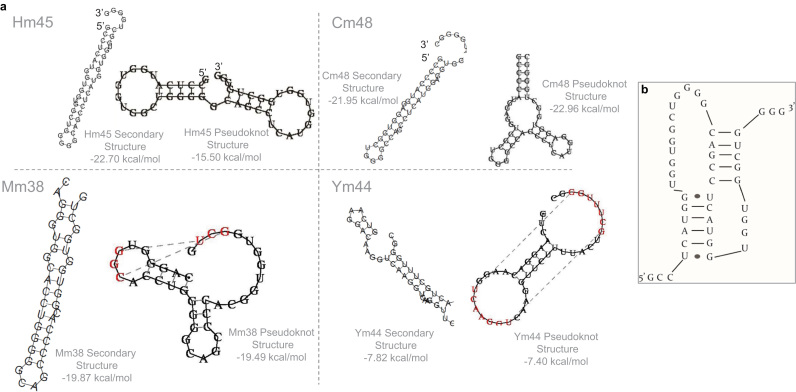
**a.** The structural variations of pseudoknots in prion protein mRNA in different species. The secondary and pseudoknot structures and the free energy were calculated by *RNAstructure* and *vsfold5* as described in. **b**. A schematic drawing of the classical pseudoknot secondary structure as described by Wills (45) in human. Normally, the pseudoknot contains two stems and three loops. The sequence of the different pseudoknots used in this study were presented in Table 2.

The shsRNAs (Fig. 1) were synthesized by the methods as described previously [28]. The three shsRNAs used in this current study are RQ 11+12, RQT157 and MNV having 197, 157 and 86 nucleotides respectively (a kind gift from Dr. Grossman, Q-RNA Inc., NY). The sequence and the structure formation energy listed in the Table 1. The sequence of pseudoknots in the prion protein mRNAs have been identified by comparative sequence analysis and pattern searching [43], [44], [45]. Pseudoknots of four different species (human, cattle, mouse, and yeast) were used in this study and the structure of human prion protein mRNA pseudoknot is shown in Fig. 2 and Table 2. The prion mRNA pseudoknots of human, cattle, mouse and yeast contain 45 nucleotides, 47 nucleotides, 38 nucleotides, and yeast 44 nucleotides respectively [43], [45]. These mRNA pseudoknots have been referred as Hm 45, Cm 47, Mm 38 and Ym 44 respectively and obtained from Sigma-Aldrich Ltd. All other nucleic acids were purchased from Sigma. The purity of the oligonucleotides was verified by electrophoresis in a 13%, polyacrylamide gel in 7 M urea that showed more than 99% homogeneity of the nucleotides. The concentrations of the nucleic acids were measured by optical methods using the molar extinction coefficients supplied by the manufacturer. All other compounds used in the buffers were of analytical grade.

**Table 1:** The sequence and thermodynamic parameters of the small-highly-structured RNAs used in this study.

**RNA**	**Sequence**	**No. of nt**	**ΔG (Kcal/mol)**	**GC Content**
RQ11+12	5′.GGGGUUUCCAACCGGAAUUUGAGGGAUGCCUAGGCAUCCCCCGUGCGUCCCUUUA	197	− 102.07	59.40%
	CGAGGGAUUGUCGACUCUAGUCGACGUCUGGGCGAAAAAUGUACGAGAGGACCUU			
	UUCGGUACAGACGGUACCUGAGGGAUGCCUAGGCAUCCCCGCGCGCCGGUUUCGG			
	ACCUCCAGUGCGUGUUACCGCACUGUCGACCC.3′			
RQ157	5′.GGGGUUUCGAACCGGAAUUUGAGGGAUGCCUAGGCAUCCCCCGUGCGUCCCUUUACGAGGGAUUGUCGACUCUAGAGGAUCCGGUACCUGAGGGAUGCCUAGGCAUCCCCGCGCGCCGGUUUCGGACCUCCAGUGCGUG UUACCGCACUGUCGACCC.3′	157	− 85.73	61.80%
MNV	5′.GGGUUCAUAGCCUAUUCGGCUUUUAAAGGACCUUUUUCCCUCGCGUAGCUAGCUACGCGAGGUGACCCCCCGAAGGGGGGUGCCCC.3′	86	− 42.92	59.30%

**Table 2:** The sequence and thermodynamic parameters of the prion mRNA pseudoknots of different species used in this study.

**Pseudoknot (nt)**	**Nucleotide sequence**	**GC content**	**Free energy (Kcal/mol)**
Hm45	5′.GCCUCAUGGUGGUGGCUGGGGGCAGCCUCAUGGUGGUGGCUGGGG.3′	71.10%	− 22.7
Cm48	5′.GCCCCAUGGAGGUGGCUGGGGCCAGCCUCAUGGAGGUGGCUGGGGC.3′	73.90%	− 21.95
Mm38	5′.CAGGGUGGCACCUGGGGGCAGCCCCACGGUGGUGGCUG.3′	76.30%	− 19.87
Ym44	5′.GUCAAGGACAAGGUCAAGGUCAAGGUUCUUUUACUGCUUUGGGC.3′	47.70%	− 7.82

The fifteen base pair GAGCTTCAAAGGGTG oligo-nuleotide binding sequence of HMG proteins and twenty eight base pair binding sequence (GACTTGTGGAAAATCTCTAGCAGTGCAT) of HIV-1 gag protein, which constitutes the stem loop region of the packaging signal and have been termed as Lef- and NC- DNA respectively were obtained from QIAGEN [49], [50], [51]. Concentrations of single strands and duplexes were determined from the A_260_ of nucleotides. DNA duplexes were prepared by mixing the complementary single stranded oligonucleotide in equimolar amounts (1:1) in 10 mM Hepes-KOH (pH 7.5) containing 100 mM NaCl, 10 mM MgCl_2_ buffer and hybridized by cooling slowly from 90 °C to 20 °C over several hours [52].

### 2.3 Steady-state fluorescence anisotropy measurements

The steady state anisotropy measurement was described earlier [49]. Briefly, the fluorescence anisotropy was measured with a Hitachi-4500 spectrofluorometer equipped with an accessory for steady-state polarization measurements. Every experiment was performed three technical replications and plotted the average values with error-bars as standard-deviations. The temperature of the measurements was maintained at 20 °C with a circulating water thermostat. The solution was excited by 280 nm light for tryptophan fluorescence and anisotropy of emission collected at 350 nm. A 10 mm cuvette was used for the fluorescence measurements. Since the structural conversion of prion protein takes place in a lower pH [53], we have studied the binding interaction between prion protein and different nucleic acids in 0.1 M acetate buffer, pH5, in addition to compare the binding results at pH7.2 (in 0.1 M Tris-HCl buffer). In both the cases a fixed concentration (0.22 µM) of recombinant prion protein was used.

To study the pH effect on PrP structure, a steady state fluorescence property of the bis-ANS in the presence of protein at different pHs was measured in a 1 cm cuvette by exciting the solution at 360 nm and recording the emission spectra between 460 and 550 nm. The fluorescence intensity of bis-ANS does not influenced by the wide range of variable pH.

### 2.4 Secondary structure of prion proteins

Secondary structure was of α-PrP, and mouse PrP121–231 fragment was monitored by circular dichroism (CD) measurements in a JASCO-810 spectropolarimeter equipped with a Peltier thermostat as described earlier [53], [54]. Spectra were collected at 50 nm/min speed, and an average of five scans were collected to obtain the final spectrum. The temperature of the experiments was kept fixed at 20 °C. Concentrations of α-PrP and the fragment PrP(121–231) were 12 µM and 28 µM respectively. All spectra were corrected by subtracting corresponding buffer spectrum.

### 2.5 Binding between prion protein and nucleic acids

We have measured the fluorescence anisotropy of the intrinsic tryptophan groups present in α-PrP as reporters (seven tryptophan groups present in its N-terminal unstructured segment) for binding study with nucleic acids with reference to the free protein in solution. Therefore these experiments could measure the changes in the size of the protein complexes whether they are bound to the nucleic acids or not. To address the proper change of fluorescence properties due to complex formation between prion protein and different RNA molecules, we used anisotropy (r), and the values are calculated as follows [55], [56]:

(1) r=(I‖−I⊥)/(I‖+2I⊥)

where I_||_ and I_⊥_ are respectively the intensities of the emitted light when the emission polarizer is aligned parallel and perpendicular to the anisotropy of the excited light.

The binding data were analyzed by the Hill binding isotherm [57] for cooperative binding [58]:

(2) logθ=αHlog([L])−logKd

where, the degree of co-operativity is characterized by the Hill coefficient (α_H_). The Hill coefficient is calculated as the slope of the Hill plot at logθ = 0. The Hill plot is a plot of logθ Vs log [L]. Where θ is defined by the following equation [58]:

(3) θ=Ȳ/(1−Ȳ)

Ȳ is the fractional saturation of the protein. For our experimental condition, Ȳ was calculated from fluorescence anisotropy (r) increase and expressed as follows:

(4) Ȳ=(r−rmin)/(rmax−rmin)

where r_min_ is the fluorescence anisotropy value without RNA and the r_max_ is the value corresponding with the plateau [53]. It is worthy to mention that the slope in the fluorescence anisotropy plot indicates the binding strengths and/or larger or smaller prion-RNA complex formation. The slope affects the dissociation constant for sure, while the amplitude does not. The binding site size (n) is derived from the extrapolation of the initial slope with anisotropy plateau in titration carried out in linear stoichiometric conditions. The co-operativity binding site size (n) is related with the Hill coefficient (α_H_) by:

(5) αH=n/(n−rmax)

## 3 Results

### 3.1 Binding of shsRNAs with α-PrP

**Fig. 3:**
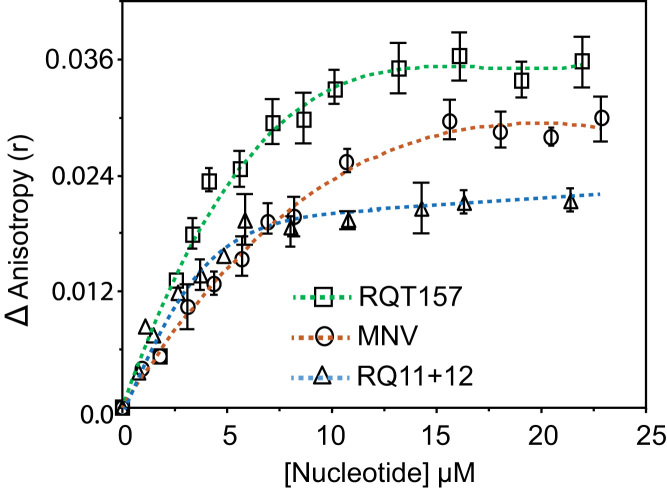
Increase in the relative tryptophan fluorescence anisotropy of α-PrP in 0.1 M Tris-HCl, pH 7.2 with the increase in the concentrations of different small highly structured RQ 11+12, RQT 157 and MNV having 197, 157 and 86 nucleotides respectively. Excitation, 280 m, emission, 350 nm. The titration experiment was performed with three replications and plotted the average values with error-bars as standard-deviations.

The prion protein in solution showed a low value for tryptophan fluorescence anisotropy (r_0_) in agreement with the presence of these groups in the highly unstructured N-terminal segment of the prion protein. The addition of increasing concentrations of shsRNAs to the protein (0.22 µM solution) in pH 7.2 buffer increased the tryptophan anisotropy values and attained a saturation value at higher nucleotide concentrations (Fig. 3). The saturation anisotropy values of the tryptophan of the protein occurred at ~ 10, 20 and 15 µM for RQ 11+12, MNV and RQT157 respectively. At the saturation, the anisotropy values for the protein in the complexes increased by more than two fold compared to the anisotropy value of the protein in buffer. The binding constants were calculated from Hill plots and the values have been presented in the Table 3.

**Table 3:** Binding data analysis for the prion protein –nucleic acid interactions.

**Nucleic acids**	**Protein**	**Ex. conditions**	**n (site size)**	**α**_**H**_	**K**_**D**_**(µM)**
RQ11+12	αPrP	pH7.2	5.8±0.62	1.4±0.61	2.2±1.5
MNV	αPrP	pH7.2	4.9±0.52	1.63±0.54	4.15±0.61
RQT157	αPrP	pH7.2	3.77±0.49	2.4±0.51	2.89±1.5
tRNA	αPrP	pH5	5.67±0.58	1.91±0.61	2.38±0.71
tRNA	αPrP	pH7.2	3.85±0.18	1.71±0.21	1.66±0.72
Hm45	αPrP	pH5	5.91±0.39	1.97±0.42	2.90±0.41
Hm45	αPrP	pH7.2	6.09±0.26	1.15±0.27	3.34±0.15
Ym44	αPrP	pH5	5.53±0.27	1.75±0.31	1.63±0.40
Ym44	αPrP	pH7.2	4.94±0.11	1.53±0.15	1.79±0.57
Cm47	αPrP	pH5	4.84±0.21	1.96±0.22	1.51±0.35
Cm47	αPrP	pH7.2	4.90±0.13	1.51±0.1	1.61±0.4
Mm38	αPrP	pH5	6.62±0.11	1.75±0.13	3.82±0.40
Mm38	moPrP	pH5	7.10±0.37	1.91±0.34	3.09±0.32
Hm45	moPrP	pH5	5.94±0.12	1.61±0.24	3.09±0.43
Cm47	moPrP	pH5	6.72±0.35	1.6±0.12	4.76±0.34
Ym44	moPrP	pH5	7.02±0.34	1.56±0.27	3.14±0.32
NC-DNA	αPrP	pH5	4.84±0.27	1.87±0.31	1.11±0.23
NC-DNA	αPrP	pH7.2	5.36±0.24	1.36±0.19	1.29±0.21
Lef-DNA	αPrP	pH5	7.32±0.51	1.08±0.45	1.25±0.32
Lef-DNA	αPrP	pH7.2	3.55±0.53	1.67±0.55	5.73±0.57

### 3.2 Binding of prion protein mRNA pseudoknots with α-PrP

**Fig. 4:**
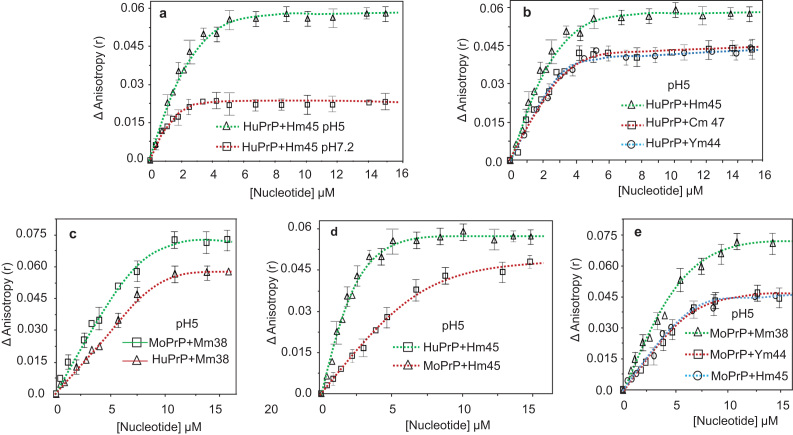
Interaction between human and mouse recombinant prion protein (α-PrP and moPrP respectively) with different pseudoknots. **a.** binding of α-PrP with human prion protein mRNA pseudoknot Hm 45 in 100 mM Tris-HCl pH 7.2 and in 100 mM acetate buffer, pH 5 measured from the increase in the fluorescence anisotropy of the tryptophan groups in the protein. Difference of anisotropy values of α-PrP in buffer and in the presence of nucleotides were plotted against nucleotide concentration. **b.** Binding of α-PrP with three different prion protein mRNA pseudoknots Hm45 (human), Cm47 (cattle) and Ym44 (yeast) at pH 5. **c.** comparison of binding interaction of mouse prion protein mRNA pseudoknots with α-PrP and moPrP at pH 5. **d.** comparison of binding of human pseudoknot Hm45 with α-PrP and moPrP. **e.** binding of moPrP with mouse, human and yeast mRNA pseudoknots at pH 5. Protein concentrations were 220 nM for all the experiments. Temperature of measurements 20 °C. Data points were fitted with a rectangular hyperbola (R^2^ is over 0.9 for all the curves) and the apparent K_D_ values are presented in Table 3. These titration experiments were performed with a set of three technical replications and plotted the average values with error-bars as standard-deviations.

The titration of the human protein by the pseudoknot region of human prion mRNA (Hm45) at pHs 7.2 and 5 showed an increase of the tryptophan fluorescence anisotropy similar to what was observed with other nucleic acids (see above). The anisotropy values were larger for binding at pH 5 and attained saturation at nucleotide to protein ratios of ~ 15 and 30 at pH 7.5 and 5 respectively (Fig. 4**a**). The anisotropy value at saturation at pH 5 was ~ two times higher compared to the value at neutral pH.

We have compared the binding of cattle and yeast mRNA pseudoknots, Cm47 and Ym44 with human α-PrP to the binding of human pseudoknot Hm45 to the protein at pH5 (Fig. 4**b**). The titration results showed similar increase in the tryptophan anisotropy values when human α-PrP was titrated by cattle and yeast pseudoknots. Although the saturation of binding of human prion protein with three different pseudoknots occurred above 6 µM nucleotide concentrations, saturation fluorescence anisotropy values resulting from binding of Hm45 to human α-PrP were ~ 20% higher. Further, slope of the tryptophan fluorescence anisotropy with Hm45 was larger to a certain extent than the same when human α-PrP was titrated by the other two mRNA pseudoknots (Figs. 4**a** and 4**b**).

To establish, if prion protein forms larger complexes with prion protein mRNA pseudoknots of the same species, we carried out binding studies of different mRNA pseudoknots with human and mouse prion proteins. The titration of mouse prion protein with mouse prion mRNA pseudoknot formed larger complex as evidenced from higher tryptophan fluorescence anisotropy value (Fig. 4**c**). Binding profiles of mouse prion protein with human and yeast mRNA pseudoknots (Hm45 and Ym44 respectively) showed decreased slopes of anisotropy with increasing pseudoknot concentrations (Fig. 4**c**). The saturation anisotropy values obtained with these pseudoknots were lower than those obtained when mouse prion protein was titrated with mouse RNA pseudoknot, Mm38 (Fig. 4c). The results shown in Figs. 4b and 4c suggest that prion protein of a species forms larger complex with the mRNA pseudoknots from the same species. This was further substantiated when human prion protein binding was studied with human and mouse mRNA pseudoknots as presented in Fig. 4**d**. The results again showed that the human prion protein with human mRNA pseudoknots forms larger complexes (higher fluorescence anisotropy value of tryptophan) on binding compared to the complexes formed by mouse prion protein with human mRNA pseudoknots (Fig. 4d). The fluorescence anisotropy at saturation showed that anisotropy values increased by 3 and 2.2 folds respectively. Similar results were obtained when human α-PrP and moPrP was bound to mouse mRNA pseudoknot (Fig. 4**e**). It needs to be mentioned that the two yeast pseudoknot sequences (Sup35p and Rnq1) do not match the human pseudoknot sequence although there is a striking similarity in pseudoknot structure which may suggest that its structure and not the sequence is important for its interaction with prion protein [43].

### 3.3 Binding of α-PrP with tRNA and PolyA

**Fig. 5:**
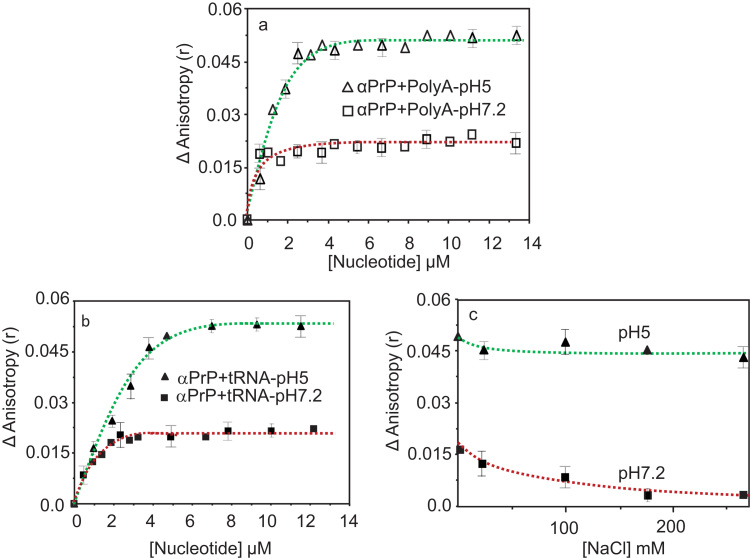
Binding interaction of α-PrP with PolyA (**a)** and tRNA (**b**) either in 100 mM acetate buffer pH 5 or in 100 mM Tris-HCl buffer pH7.2. Experiments were carried out as mentioned earlier. **c.** The effect of salt on the interaction between α-PrP and tRNA at pH 7.2 and 5. The effect of NaCl on the complex formed between α-PrP (0.22 µM) and tRNA (12 µM) when saturation values of fluorescence anisotropy were attained in buffers. Small aliquots of 4 M NaCl were added to the solution to attain the desired NaCl concentrations. Temperature 20 °C. All these titration experiments were performed with a set of three technical replications.

The saturation of titration occurred at nucleotide to protein ratio of ~ 15 and ~ 30 at pHs 7.2 and 5 respectively when α-PrP was titrated with PolyA (total nt is 910). There was ~ 2.8 fold increase in the fluorescence anisotropy value of tryptophan at pH5 which was ~ 1.6 times of the same obtained at neutral pH (Fig. 5**a**). The addition of tRNA (80 nt) to the prion protein increased fluorescence anisotropy (Fig. 5**b**) of its tryptophan groups as observed for the shsRNAs. At saturation, the fluorescence anisotropy of the tryptophan increased by 1.6 fold at pH 7.2 whereas the increase was > 2.5 fold when the protein was titrated with tRNA at pH 5. A comparison shows that at saturation, among nucleic acids studied here, the tryptophan fluorescence anisotropy value was maximum when α-PrP binds to the shsRNA RQT 157 at neutral pH (Fig. 3).

### 3.4 Salt effects on the binding interaction between prion protein and nucleic acid

Previously we found that prion protein-DNA interaction was perturbed by increase in NaCl concentrations in the buffer (unpublished results). Here we report the effect of increasing concentrations of NaCl on the binding interaction between human α-PrP and tRNA at pHs 5 and 7.2 (Fig. 5c). The presence of increasing concentrations of NaCl in buffer at pH 7.5 prevented the formation of human α-PrP complex with tRNA as evidenced from the reduced values of tryptophan anisotropy (Fig. 5**c**). The dependence of interaction producing prion protein-tRNA complex is nonlinear and has a tendency to saturate above 250 mM salt concentration. There was a decrease in tryptophan anisotropy value with the increase with NaCl concentration. Similar experiments at pH 5 showed that tryptophan fluorescence anisotropy decreased in marginal amount (Fig. 5c). These results indicate that although electrostatic interaction is dominant in the prion protein-tRNA interaction at neutral pH, forces other than electrostatic play a dominant role in the protein–tRNA interaction in acidic pH. Similar results were obtained with prion protein and DNA interaction [53].

### 3.5 Secondary structure of the prion protein

**Fig. 6:**
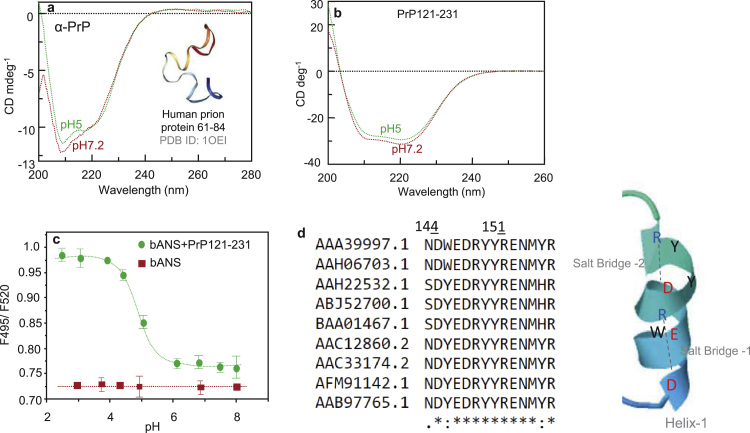
Circular dichroism spectra of α-PrP (**a**) and moPrP 121–231 fragment (**b**) in Tris-HCl pH 7.2 (red) and acetate buffer pH 5 (green). in the panel **a**, *in set*, represents the structure (PDB ID: *1OEI*) of pH-dependent folding of octapeptide repeats region of human prion protein. The result shows that the secondary structures of the full-length prion protein and the globular fragment of the protein do not change a significant extent from neutral to pH 5. **c**, the exposure of nonpolar groups from the interior of globular structure of prion protein in acidic pH detected by a hydrophobic binding fluorescent probe bis-ANS. The fluorescence intensity of bis-ANS is pH independent. Spectra were taken after incubation of bis-ANS (8 µM) with moPrP121-231(2 µM) for 30 min at the pHs indicated in the figure. From these spectra, ratios of fluorescence intensities at 495 nm (indicative of the dye binding to the newly exposed hydrophobic groups from the interior of the protein) to the fluorescence intensity at 520 nm (for protein in buffer) has been plotted. **d**. The prion protein sequence alignment of various mammalian species, indicating a highly conserved sequence in helix-1 region (*left panel*). There are also two salt bridges present in the helix − 1 (*right panel*). A schematic diagram is presented in the right hand panel, indicating the formation of bridge between (electrostatic interaction and hydrogen-bond) arginine (R) and aspartic acid (D).

The secondary structure was studied by far UV circular dichroism spectrum at 20° in 0.1 M Tris-HCl buffer pH 7.2 and 0.1 M acetate buffer pH 5 using a 12 µM α-PrP solution. The spectra show two characteristic α-helix peaks at 221 and 208 nms at neural pH (Fig. 6**a**). The nature of spectra, the positions of the peaks, and their CD intensities at pHs 5 and 7.2 were similar. The truncated mouse prion protein fragment moPrP121–231 (28 µM) which has nearly 95% sequence homology with the corresponding human prion protein fragment also showed the characteristic α-helical peaks and the spectra at pH 7.2 and 5 are practically identical (Fig. 6**b**).

### 3.6 Exposure of the nonpolar groups from the protein interior at pH 5

The previous study performed with truncated 90–231 human prion protein or PrP121–231 showed conservation of α-helical structure [47], [53]. In this present study we have performed the similar experiment with both full-length and fragment protein with all structural elements. The different pH maintaining buffer solutions containing moPrP 121–231 fragment and bis ANS were incubated for 30 min after which the spectra were recorded between 460 and 550 nm by exciting the solutions at 360 nm. The dye in the presence of the protein fragment at pH 7.2 showed an emission maximum at 520 nm (not shown). The emission spectra showed a blue shift going from neutral to acid pH. A plot of the ratio of the fluorescence intensities at 490 nm to that at 520 nm against pH of solutions ranging between 8 and 3 has been shown in Fig. 6**c**. The graphical representation shows a structural transition of the prion protein fragment occurred with the decrease pH of the solution, and the apparent structural transition occurred at pH5. A similar pK value has been obtained with the human prion protein 90–231 fragment during its structural change from neutral to acidic pH [47].

## 4 Discussion

Previously, we observed that both the single and double stranded nucleic acids induce polymerization of recombinant full-length prion protein in solution to prion protein oligomers and amyloids [31]. We have shown polymerization of prion protein to linear and spherical amyloids induced by PolyA and tRNA at pH5 [31]. Besides, the DNA condensation induced by prion protein is sequence dependent [32]. In this study, we have characterized the interaction between prion protein and different RNAs that leads to the protein polymerization process.

The present results show that α-PrP forms complexes with different RNAs having diverged base compositions with apparent binding affinities ranging from 10^5^ to 10^6^ (based on nucleotide concentrations). The binding constants vary from 3.5 to 5.8 µM and from 4.8 to 7.32 µM at pH 7.2 and 5 respectively (Table 3). Although gel electrophoresis experiments showed specificity of some shsRNA in binding to the prion protein, the present result could not identify such specificity [28]. The specificity reported earlier probably arose from some unknown factors present in the serum when the human recombinant prion protein was incubated with the shsRNAs. The binding between the protein and the nucleic acid is characterized by a small positive cooperativity at both the pHs presented in Table 3 where the stoichiometry values of a protein molecule binding to the number of nucleotides are also presented. The binding sites are independent of the nature of nucleic acids similar to the characteristics of affinity constant as observed above.

Prevention of complex formation between prion protein and tRNA by increasing concentrations of NaCl would suggest that electrostatic interaction dominates the binding interaction between these molecules at neutral pH. We consider that in these solutions, the primary NH_2_ group in the N-terminal of the protein, Lys amino groups and Arg groups are probably involved in the electrostatic binding of the protein with nucleic acid. The present data do not permit to assess the relative contribution of these groups towards this binding. Studies of prion protein binding with different DNAs show that their binding is also dominated by electrostatic interaction at neutral pH [53]. We believe that similar interaction determines binding between prion protein and shsRNAs and mRNA pseudoknots studied here at neutral pH.

Larger nucleoprotein complexes are formed at pH 5 compared to the complex at the neutral pH for all the RNAs studied here. However, the presence of NaCl has much less effect in the nucleoprotein complex formation between tRNA and prion protein at pH 5 (Fig. 5c). This would suggest that forces other than electrostatic play an important role for binding of the protein and tRNA at pH 5. A possible explanation of the stability of prion protein-tRNA complex in NaCl at pH 5 could arise from the interaction of nucleic acid with the nonpolar groups of prion protein exposed from the α-helical (particularly helix-1) segment of C-terminal 121–231 amino-acids region at pH 5 (Fig. 6).

The secondary structure of α-PrP remains practically unaltered between pH 7.2 and 5 (Fig. 6*a*). The protein contains the hydrophobic domain VAGAAAAGAVV spanning 112–122 amino acid residues in the N-terminal unstructured region of the protein and these groups are expected to be exposed to the surrounding solvent in both the pHs. Since NaCl can prevent the binding between the protein and nucleic acid at neutral pH, we consider that most likely this hydrophobic region is not involved in interaction with nucleic acid in this solution. The results with the moPrP 121–231 fragment shows that the secondary structure of this protein fragment also does not change between pH 7.2 and 5 similar to what was observed for the full length protein (Fig. 6**b**). However bis-ANS experiments detect exposure of the nonpolar groups of the protein to the solvent in this pH (pH5) (Fig. 6**c**). We consider that the newly exposed non-polar groups in the surface of the full-length protein, similar to the 121–231 fragments at pH 5, would make these groups available to interact with nucleic acid that would be insensitive to salt. The newly exposed non-polar groups from the prion protein interior at pH 5 are expected to bind to the nucleic acid bases by dispersion interaction [53], [59]. We believe that similar interaction of the newly exposed hydrophobic groups from the interior of prion protein with nucleic acids is responsible for the formation of larger nucleoprotein complex with shsRNAs and prion protein mRNA pseudoknots at pH 5. In this regards, it is also questionable that the particle formations leading to light scattering may also contribute to the fluorescence anisotropy or anisotropy, particularly at the low pH. However, similar experiments were performed to determine the interaction parameters between prion protein and nucleic acids or PrP with heparin [54], [60]. The light scattering does not interfere with the anisotropy or anisotropy as the concentration of the prion protein used in these experiments were very low [54], [60]. Indeed, It is important to note that the prion protein contains seven tryptophan moieties in its most non-structural and flexible N-terminal domain. The fluorescence intensity would be high even in a low concentration of the protein; however, the anisotropy value would be lower due to the high local motion.

We observe that binding of prion protein to the shsRNAs, prion protein mRNA pseudoknots and tRNA show comparable affinities in solution and binding does not depend significantly upon pH of the solution (Table 3). Our unpublished results also show comparable binding affinities of prion protein with DNAs. Prion protein shows DNA strand transfer and RNA binding and chaperoning properties characteristic of HIV1 retroviral nucleocapsid protein, NCp7 [61]. It is also worth noting that the CUGGG motif in the human prion mRNA pseudoknot was also found in the loop of HIV TAR RNA [43]. In solution, α-PrP was found to cause the hybridization of Tar(+) to Tar(-) in a dose-dependent manner very similar to NCp7. From all these observations it has been suggested that binding energy does not determine the functional properties of the protein NCp7 in vitro. A similar conclusion may be applicable to the prion protein, which mimics binding properties of NCp7 with different nucleic acids.

It is worth noticing that the pseudoknots of prion protein mRNAs form larger complexes with the prion protein from the same species at both neutral and acidic pHs. Although the species-specific prion protein and pseudoknots interaction is suggested, but we are not certain about its significance at present. The larger complexes between prion protein and nucleic acids at pH 5 result from the binding of the nucleic acids to the newly exposed nonpolar groups from the protein interior (without any change in the secondary structure of the protein, see above). However, if we extend our reasoning that the formation of larger complexes of prion protein with the prion mRNA pseudoknots from the same species might result from an increased extent of exposure of the nonpolar groups from the protein interior in the presence of this mRNA. This would suggest that prion protein mRNA pseudoknots could destabilize the structure of prion protein of the same species to a greater extent compared to the other pseudoknots. Alternatively the explanation of the larger complexes might arise from the specific structure of pseudoknot domain. This may be indicated from the comparable binding properties of the human and yeast prion protein mRNA pseudoknots with the full-length mouse prion protein (Fig. 4) where the pseudoknot structures are identical although the base sequences of these two pseudoknots are different [43] (Table 2).

**Fig. 7:**
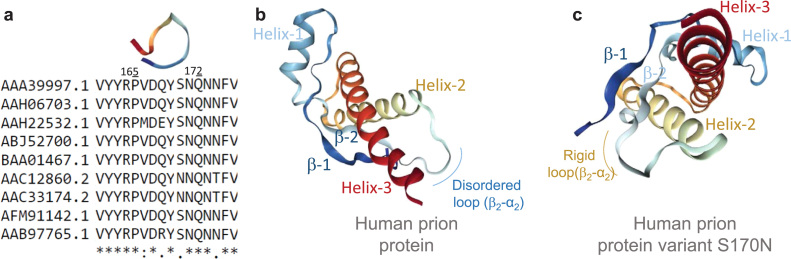
The amino acid sequence alignment at β_2_-α_2_ loop region (panel-**a**) and three-dimensional (3D) structure of full-length human prion protein. It was assumed that the β_2_-α_2_ loop region is extremely flexible and this loop region varies substantially between species. The structure also influenced by the residue types in the 2 amino acid sequence positions 170 (S or N) and 174 (N or T). The corresponding full-length α-PrP 3D structure with dis-ordered-loop (panel-**b**), and S170N mutated rigid-loop variant (panel-**c**) (PDB ID: *1QLZ* and *1E1S* respectively).

Indeed, this pH regulated prion protein –RNA interaction and species specific variable complex formation between prion protein and its mRNA pseudoknots is complex. The origin of differential interaction is probably multidimensional. It is possible that the GC content and RNA structure (secondary or pseudoknots) stability determined by formation free energy, may play a critical role in these prion protein- RNA interaction. The previous findings including our unpublished data (manuscript under review) indicated that nucleic acid with a sequence dependent (based on GC content of nucleic acids) manner triggers the conversion of cellular prion protein into the beta-sheet conformation [26]. In this regards, we tried to correlate the GC content with the free energy of secondary structures of prion pseudoknots (Table 2). The data indicated that the lowest free energy is corresponding to least GC content in the Yeast prion pseudoknots. However, within the mammalian pseudoknots it varies differently. Therefore, the interacting RNA sequence, the corresponding secondary structure and its stability may play a critical role during interaction with cellular prion protein. Besides, this species specific interaction may also control by the prion protein sequence and the corresponding three dimensional structure (Fig. 7). There are few structural features play a critical role during pH dependent higher order complex formation. At acidic pH, particularly pH5, there are two important structural transitions occurred. First, the octapeptide (amino acid residues 62–84) region in N-terminal domain turns into highly flexible; whereas, at neutral pH it shows relatively rigid structure (Fig. 6a) [62]. Besides, the salt bridge in helix-1 also dissolved and the more hydrophobic domains exposed towards solvent [53], [59]. These two structural transitions play important role during pH dependent formation of the higher order complexes. Our current data also indicate that there is certain structural transition occurred in both full-length and C-terminal domain of the prion protein (Fig. 6a-c). However, most important structural feature resides at the β_2_-α_2_ loop region. This particular region plays a critical role to control interspecies prion disease transmission [63]. The loop/turn is extremely flexible and this loop region varies substantially between species. The structure also influenced by the residue types in the 2 amino acid sequence positions- 170 (S or N) and 174 (N or T) [63], [64], [65]. The sequence alignment of prion protein amino acid sequences over the multiple species within mammalians indicate the presence of S170N variants (Fig. 7, left panel). The wild type human PrP^C^ loop is disorder, however, the PrP^C^ variant S170N leads to rigid structure (Fig. 7). The cross-species transmission occurred by a 170 S/N polymorphism. Therefore, it is possible that the interaction between specific pseudoknot with cellular prion protein may regulated by β2-α2 loop structure and eventually leads to prion transmissibility between different species.

It is important to note that the prion protein interacts with total RNA extracted from neuroblastoma cells (N2aRNA) with nanomolar affinity, and which is toxic to cultured cells. However, the small RNAs bound to PrP give rise to nontoxic small oligomers [66]. Besides, another recent study also show the similar type of findings with P53 –RNA interaction, where p53 core domain (p53C) forms prion aggregation in the presence of RNA. The result also demonstrated that RNA can modulate the aggregation of p53C and full-length p53 [67]. The role of a scrapie specific nucleic acid in prion infection has not been demonstrated yet. Evidence from the literature shows that in prokaryotic system, nucleic acid-binding proteins can bind to the specific nucleic acids in the cytoplasmic pool of non-specific nucleic acids for manifestations of their biological functions [68], [69], [70], [71]. it is generally accepted that nucleic acid in cytoplasm can catalyze the conversion of cellular prion protein to its infectious isoform [38]. The current and previous data suggested the presence of pseudoknots in the mRNAs of PrP^C^ genes, their actual folding and their possible involvement in and interference with PrP^C^ translation. Our current data is suggesting that these pseudoknots could be involved in the conformational changes of PrP^C^ to PrP^Sc^, which occurs at the endoplasmic reticulum, where translation occurs. It is possible that too many copies of pseudoknot–protein complexes could form and interfere with translation, leading to folding of PrP^C^ into its pathogenic form.
