# Supplementary material for: A quantitative characterization of interaction between prion protein with nucleic acids
Source: Biochem Biophys Rep. 2018 May 2;14:114–24. doi: 10.1016/j.bbrep.2018.04.006 (PMC5986701; doi:10.1016/j.bbrep.2018.04.006)
Supplement: Supplementary file 1 — Supplementary material [file mmc1.pdf]

CONFLICT OF INTEREST DISCLOSURE.

Dr. Alakesh Bera, PhD

Dr. Sajal Biring, PhD

Title: A quantitative characterization of interaction between prion protein with nucleic acids

Conflict of Interest disclosure.

The authors declare no conflict of interest.
